# Evaluation of deep learning models for quality control of MR spectra

**DOI:** 10.3389/fnins.2023.1219343

**Published:** 2023-08-29

**Authors:** Sana Vaziri, Huawei Liu, Emily Xie, Hélène Ratiney, Michaël Sdika, Janine M. Lupo, Duan Xu, Yan Li

**Affiliations:** ^1^Department of Radiology and Biomedical Imaging, University of California, San Francisco, San Francisco, CA, United States; ^2^Univ Lyon, INSA-Lyon, Université Claude Bernard Lyon 1, UJM-Saint Etienne, CNRS, Inserm, CREATIS UMR 5220, Lyon, France; ^3^UC San Francisco/UC Berkeley Graduate Program in Bioengineering, San Francisco, CA, United States

**Keywords:** MR spectroscopy, convolutional neural network, random forest, quality control, machine learning

## Abstract

**Purpose:**

While 3D MR spectroscopic imaging (MRSI) provides valuable spatial metabolic information, one of the hurdles for clinical translation is its interpretation, with voxel-wise quality control (QC) as an essential and the most time-consuming step. This work evaluates the accuracy of machine learning (ML) models for automated QC filtering of individual spectra from 3D healthy control and patient datasets.

**Methods:**

A total of 53 3D MRSI datasets from prior studies (30 neurological diseases, 13 brain tumors, and 10 healthy controls) were included in the study. Three ML models were evaluated: a random forest classifier (RF), a convolutional neural network (CNN), and an inception CNN (ICNN) along with two hybrid models: CNN + RF, ICNN + RF. QC labels used for training were determined manually through consensus of two MRSI experts. Normalized and cropped real-valued spectra was used as input. A cross-validation approach was used to separate datasets into training/validation/testing sets of aggregated voxels.

**Results:**

All models achieved a minimum AUC of 0.964 and accuracy of 0.910. In datasets from neurological disease and controls, the CNN model produced the highest AUC (0.982), while the RF model achieved the highest AUC in patients with brain tumors (0.976). Within tumor lesions, which typically exhibit abnormal metabolism, the CNN AUC was 0.973 while that of the RF was 0.969. Data quality inference times were on the order of seconds for an entire 3D dataset, offering drastic time reduction compared to manual labeling.

**Conclusion:**

ML methods accurately and rapidly performed automated QC. Results in tumors highlights the applicability to a variety of metabolic conditions.

## Introduction

MR spectroscopy (MRS) is a valuable tool to measure *in vivo* information of cellular metabolism, thus enabling noninvasive monitoring of metabolic changes due to disease progression, therapeutic response, and treatment effects. Numerous research studies have highlighted its applicability to a variety of diseases, such as cancers, neurodegeneration, developmental disorders, and ischemic injuries (Preul et al., 1996; Nelson et al., 1999; Carhuapoma et al., 2000; Kurhanewicz et al., 2000; Schuff et al., 2006; Bejjani et al., 2012; Li et al., 2015b). While most clinical applications favor single voxel acquisitions due to the simplicity in interpretation and limited data, multi-voxel acquisition, also called MR spectroscopic imaging (MRSI), improve signal-to-noise ratios (SNR), spatial coverage, and provide flexibility in acceleration. Recently, active efforts in high field MR and significant advances in both accelerated acquisitions and post-processing signal enhancement methods have greatly improved the new generation of whole-brain, high-spatial resolution MRSI (Nelson et al., 2013; Bogner et al., 2021).

Accurate metabolite concentration quantification depends on the spectral quality of the voxel. SNRs of metabolites, linewidths of peaks, and quantitative error estimates such as Cramér-Rao lower bounds, which are derived from metabolite fitting to describe errors in the computed metabolite concentrations, are typically used to determine spectral quality (Jiru et al., 2006; Oz et al., 2014; Wilson et al., 2019; Maudsley et al., 2021). However, additional quality control (QC) is still required to filter artifacts such as lipid contamination and inadequate water suppression, which are typically performed manually through voxel-wise visual inspection. For a single 3D dataset, hundreds of voxels are individually reviewed by MRS experts to identify and exclude those of poor quality or containing artifacts. This time-consuming process poses a severe hindrance to the adaptation of 3D MRSI to clinical and translational studies.

Recently, machine learning (ML) approaches, which can quickly triage large amounts of imaging data, have also been applied for spectral QC. Menze et al. introduced the use of a random forest (RF) classifier trained on magnitude spectral data to label spectral quality based on patterns in the spectra (Menze et al., 2008). This method achieved an AUC of 0.950 for voxels within the acquisition volume and, when compared to human experts, outperformed the use of decision rules based on SNR and Cramér-Rao-bound estimates derived from spectral fitting. Rather than using the magnitude spectra as input, RF classifiers were later evaluated in patients with glioblastoma using spectra-derived parameters (Tensaouti et al., 2022), resulting in an AUC of 0.955. Similarly, Wright et al. proposed the use of a support-vector machine that was trained on features that were first extracted using independent component analysis on the spectra (Wright et al., 2008). Pedrosa de Barros et al. then used a RF classifier trained on both time-domain and frequency-domain features to perform QC and achieved an area under the curve (AUC) of 0.998 (Pedrosa de Barros et al., 2016). Apart from the RF classifier proposed by Menze et al. (2008), the ML methods described work by first extracting features from the spectral data. The ML models for automated classification were then trained using the extracted features as input.

Although the RF model proposed for QC has the advantage of ease of implementation and few hyperparameters to be selected, recent work has highlighted the potential of improvements with more complex neural network models. In such methods, rather than relying on a set of features derived through fitting algorithms or spectral decomposition, the spectral waveform is used as the direct input to a deep neural network. Meaningful features can then be implicitly learned during model training via hidden layers of deep networks. A large and diverse dataset covering the variance seen in real data is required for training such complex models. Kyathanahally et al. successfully applied fully connected neural networks (FCNNs) and convolutional neural networks (CNNs) to the complete 2D time-frequency spectrogram representations of raw 1D spectra to identify and remove ghosting artifacts on simulated and healthy volunteer datasets (Kyathanahally et al., 2018). Gurbani et al. developed a CNN model for QC labeling which takes as input the spectral waveforms pre-filtered by linewidth directly and achieved an AUC of 0.951 when evaluated on 9 patients with glioblastoma (Gurbani et al., 2018). Despite the small sample size and limited scope of disease, these studies demonstrated the potential of using ML-based methods for automatic 3D MRSI QC for clinical applications without the need for feature engineering.

In this study, we evaluated the performance of deep neural networks in comparison to the RF classifier for QC of short-echo 3D MRSI datasets collected from patients with various neurological diseases. Using expert classifications of spectra as voxel-wise labels, five ML approaches were trained and evaluated. These models circumvent the need for both feature engineering and spectra filtering based on metabolite linewidth and SNR as they all take as input the complete spectra. A simple RF classifier was first evaluated (Menze et al., 2008) and compared to a 6-layer CNN (Gurbani et al., 2018). Then, the introduction of more hidden layers to the model architecture to represent higher-order features was explored via the more complex inception CNN (ICNN) module (Szegedy et al., 2015). Finally, we evaluated the use of the two deep learning models (CNN and ICNN) to determine abstract features that were then used as input to the RF classifier. In these hybrid methods (CNN + RF, ICNN + RF), the DL-derived features are used forwarded as input to the RF classifier. All 5 classifiers (RF, CNN, ICNN, CNN + RF, ICNN + RF) were trained and evaluated on data acquired from healthy volunteers, patients with neurological disorders (including major depressive disorder, multiple sclerosis, and Parkinson’s disease), and patients with brain tumors.

## Materials and methods

### 3D MRSI data and imaging

A total of 53 7 T MR datasets from prior studies [10 from healthy controls, 10 from patients diagnosed with major depressive disorder (Li et al., 2016), 10 from patients with multiple sclerosis (Henry et al., 2015), 10 from patients with Parkinson’s disease, and 13 from patients with brain tumors (Li et al., 2015a)] were retrospectively analyzed after appropriate approval from our Institutional Review Board and informed consent from subjects. Data from patients with Parkinson’s disease with low quality spectra was included in the analysis to balance the ratio of good to bad quality spectra during ML training. These datasets are hereafter referred to as the ND dataset (all data obtained from healthy controls and patients with neurological disorders, *N* = 40) and the BT dataset (all data obtained from patients with brain tumors, *N* = 13).

MR data were obtained on a GE 7 T MR950 scanner (GE Healthcare, Waukesha, WI). 3D MRSI datasets were acquired with TE = 30 ms (BT) or 20 ms, TR = 2,000 ms, 1 cm isotropic spatial resolution, and (18–20) × 22 × 8 matrix size (Henry et al., 2015; Li et al., 2015a, 2016). Anatomic images included 3D T1-weighted inversion recovery-prepared spoiled gradient echo (IRSPGR) images [TR/TE/ inversion time (TI) = 6/2/600 ms, matrix size = 256 × 256 × 192, FOV = 256 × 256 × 192 mm^3^] and 2D T2-weighted fast spin echo [TR/TE/TI = 6,000/86/600 ms, matrix size = 512 × 512, field of view = 241 × 241 mm^2^, 19–21 slices, slice thickness/gap = 3/1 mm]. For each BT dataset, an additional MR examination was performed on a 3 T MR750 scanner (GE Healthcare, Waukesha, WI), which included T2-weighted fluid attenuated inversion recovery (FLAIR) images (TR/TE/TI = 6,250/139/1,699 ms, slice thickness = 1.2 mm, FOV = 25.6 × 25.6 cm, matrix = 256 × 256) and T1-weighted ISPRGR images (TR/TE/TI = 6.6/1/450 ms, slice thickness = 1.5 mm, field of view [FOV] = 25.6×25.6 cm, matrix = 256 × 256). T2 hyperintense lesions (T2L) were identified manually on the 3 T FLAIR images which were then rigidly registered linearly to 7 T T1 images using FLIRT (Jenkinson et al., 2012, FMRIB Software Library, fsl.fmrib.ox.ac.uk/fsl/fslwiki/FSL). The registration transformation matrix was applied to the T2L masks to convert to 7 T space. The T2L masks were down-sampled to the MRSI resolution and voxels containing any overlap (>0%) with the high resolution T2L ROI were classified as tumor (Li et al., 2018).

### QC manual labeling

The 3D MRSI datasets were reconstructed and processed as described previously (Li et al., 2015a). Prior to metabolite quantification, the coil-combined and frequency and phase corrected spectra within the excitation region were labeled for quality independently by two experts. Both experts had over 15 years of experience evaluating brain metabolism with MRS. Manual labeling took on the order of 10–15 min per dataset and was based on subjective evaluation of SNR, linewidth, presence of lipid artifact, and incomplete water suppression. Following the conservative approach taken in Menze et al. (2008), voxels were labeled “good” if both raters labeled it as such. Voxels labeled as poor quality by either rater was labeled as “bad.” The final aggregated ND data set consisted of 9,030 voxels labeled as “good” and 7,723 as “bad.” The aggregated BT dataset consisted of 3,863 voxels labeled as “good,” 1,548 as “bad.” Of these voxels, 1,053 were within the T2L (684 “good,” 369 “bad”).

### Pre-processing of spectral data for model input

Voxel-wise real-valued spectra were extracted from complex signals and cropped to 1.4 to 4.1 ppm (850 spectral points). Cropped spectra were normalized to have mean 0 and standard deviation 1 for input to ML models.

### Network architectures

**Figure 1:**
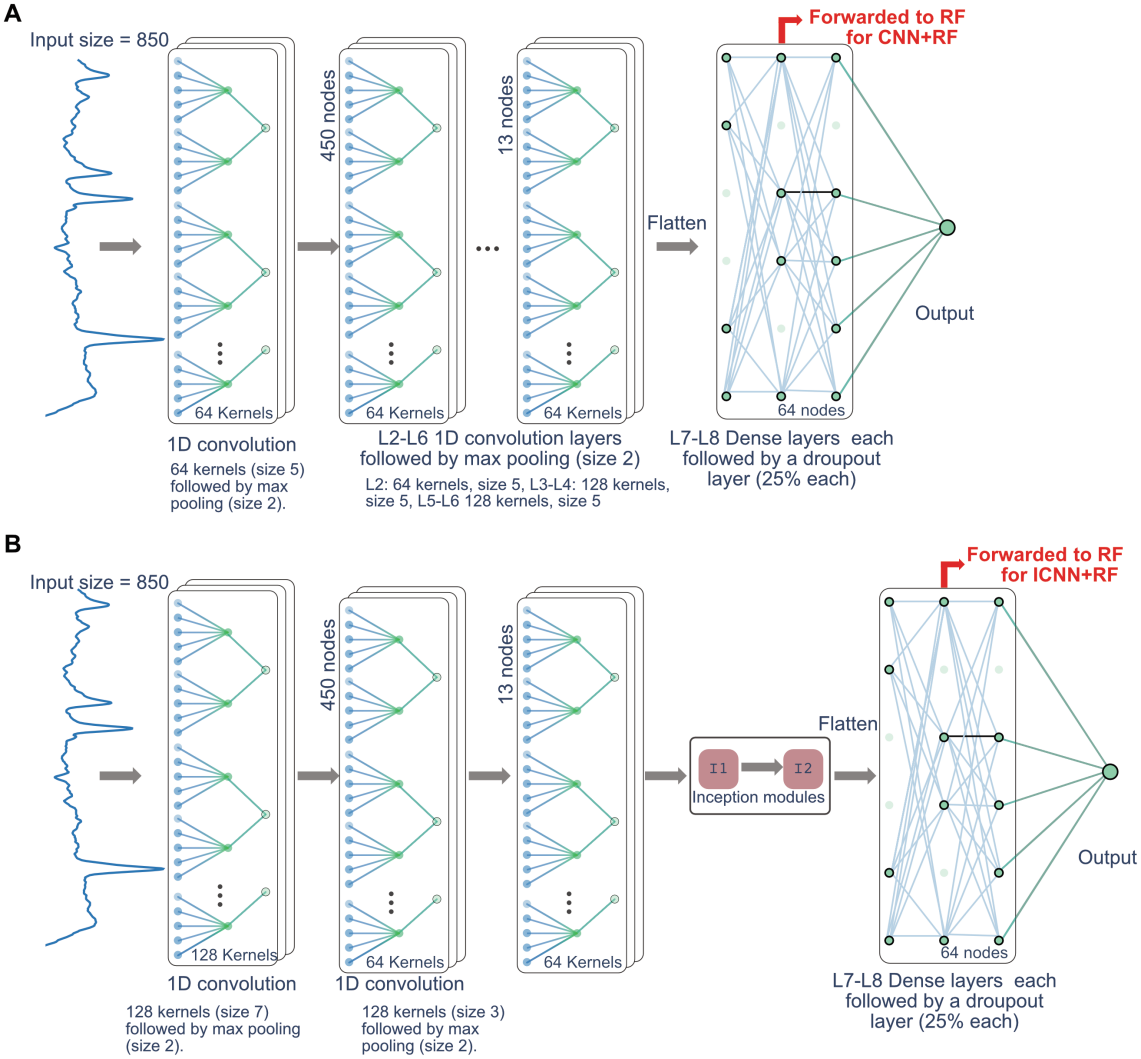
Network architecture for CNN model **(A)** and ICNN model **(B)**. The nodes forwarded to RF for the CNN + RF and ICC + RF models are indicated by the red arrow.

All computational work was performed on a custom-built workstation with AMD 3900× 12-core CPU with a Nvidia Titan X GPU, utilizing Python 3.8 and Tensorflow 2.2. Three base models were constructed using: (1) an RF classifier, (2) a standard CNN, and (3) an inception CNN (ICNN). For all models, the 850-point normalized real-valued spectra was used as input. The RF classifier was built using 200 estimators and the standard number of features to grow each tree for classification [$\left(\sqrt{850}\right)\approx 30;$ Liaw and Wiener, 2002]. The CNN was modeled as a tile-free modification of the network in Gurbani et al. (2018) and consisted of 6 convolution layers with max pooling, 2 fully connected layers. The ICNN consisted of 2 convolutional layers with max pooling, 2 inception module layers (Szegedy et al., 2015) followed by max pooling, 2 fully connected layers, and a final output layer. The CNN and ICNN are depicted in Figure 1. Finally, the CNN and ICNN were combined with the RF classifier to build two additional hybrid models as follows: the CNN + RF and ICNN+RF models were created by first training the CNN and ICNN and then extracting the features generated prior to the final output layers (64 nodes for both CNN and ICNN). These nodes were then used as input nodes to an RF classifier, which produced the final QC prediction. Hybrid model input nodes are indicated by the red arrows in Figure 1.

### Model training and evaluation of prediction accuracy

For the ND dataset, each model was cross-validated by reserving data from 4 subjects for testing model (one of each type: healthy volunteer, major depressive disorder, multiple sclerosis, and Parkinson’s disease). Another 4 subjects were similarly reserved for model validation to prevent any leakage. Voxels from the remaining datasets were aggregated and used for training. This was repeated a total of 10 times using a different set of patients for the validation and testing sets and results in an approximate 75%/12.5%/12.5% split of voxels in the training/validation/test datasets. For the BT dataset, cross-validation was performed using leave-one-out analysis for each of the 13 patient dataset. The validation set was made of two randomly selected patients to prevent leakage. Voxels from the remaining datasets were aggregated and used for training. This results in an approximate 75%/12.5%/12.5% split of voxels in the training/validation/test datasets. For the CNN and ICNN, the Adam optimizer was used with the categorical cross-entropy error of class labels to output probabilities and the initial learning rate was set to 1e − 4 (Kingma and Ba, 2014; Gurbani et al., 2018). Models were trained with a batch size of 64 and with 15 epochs. Model AUC (i.e., ROC-AUC) and accuracy were evaluated on the test datasets. The AUC of the precision-recall curve (AUC-PR) was also calculated (Buckley and Voorhees, 2000; Davis and Goadrich, 2006). For the BT dataset, each model was further evaluated based on AUC, AUC-PR, and accuracy calculated using T2L voxels only. To evaluate the importance of spectral regions on DL results, the method of integrated (IG) gradients (Sundararajan et al., 2017; Wargnier-Dauchelle et al., 2021) was used to visualize feature importance for the base models in tumor lesions.

## Results

### The ND dataset

**Figure 2:**
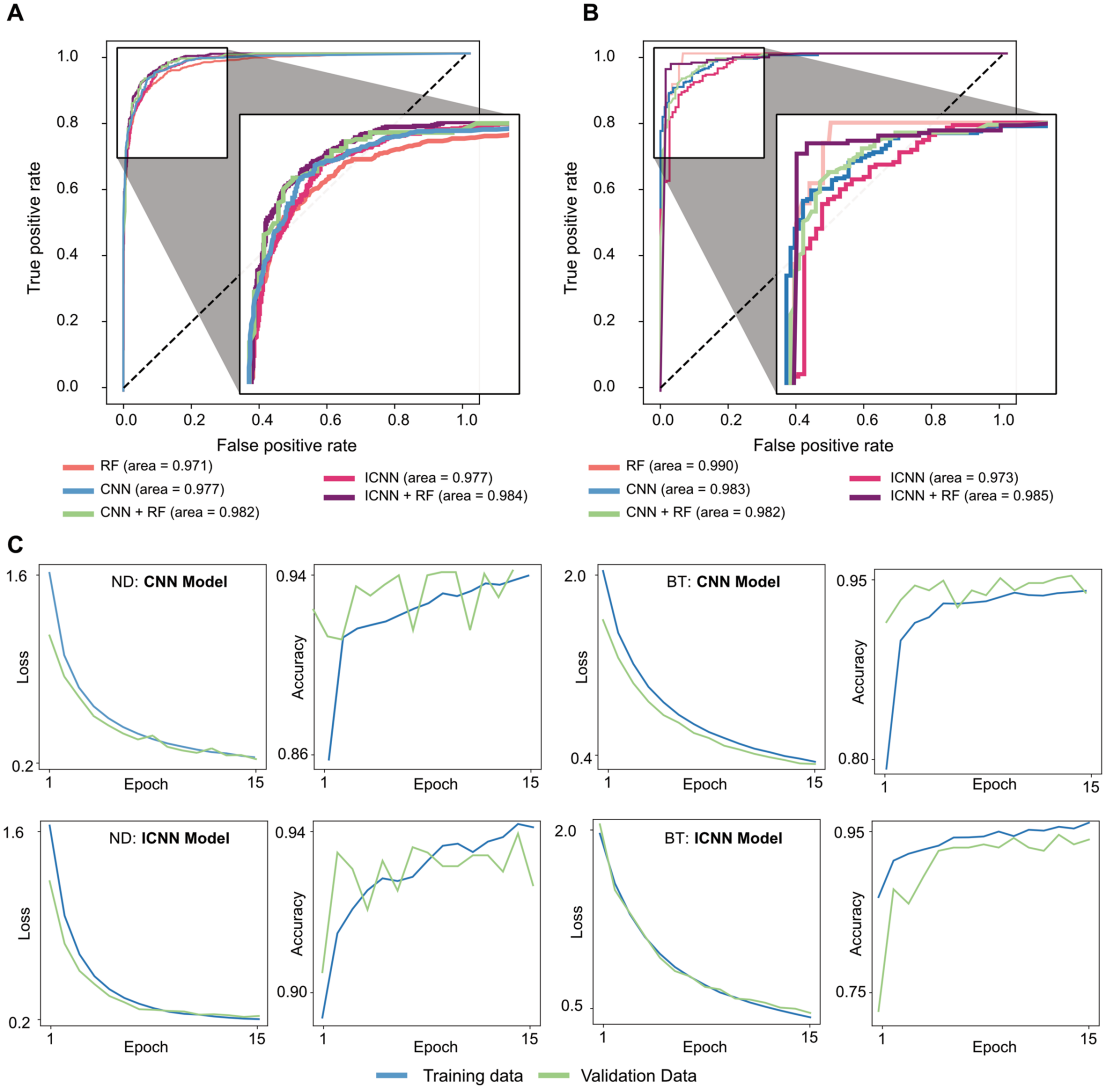
Sample ROC curves for all models trained on **(A)** ND dataset and **(B)** BT dataset, and Sample CNN and ICNN loss and accuracy evaluated in training (blue) and validation (green) sets **(C)**.

**Figure 3:**
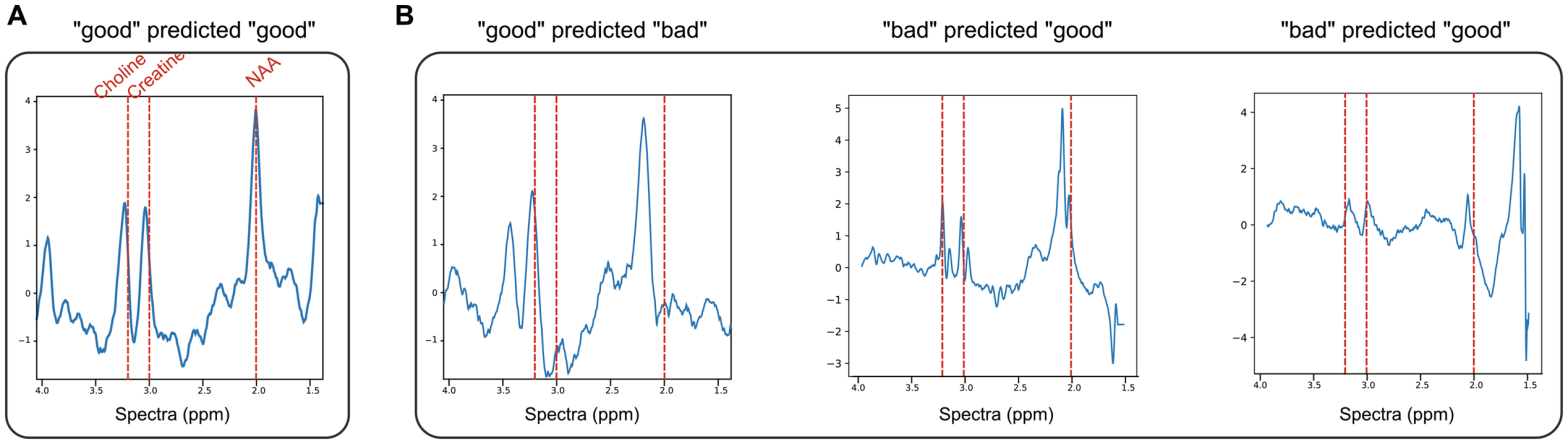
ND dataset examples of normalized spectra with data quality predictions using CNN + RF. **(A)** A representative “good” voxel that was correctly predicted as “good” using the CNN + RF. **(B)** Example of mispredicted voxels using the CNN + RF: a voxel labeled “good” but predicted “bad” (left) and two voxels labeled as “bad” but predicted “good” (middle, right).

The model training times, AUC, AUC-PR, and accuracy are given in Table 1, and sample ROC curves for all models along with CNN and ICNN training/validation loss and accuracy curves are illustrated in Figure 2A. Average prediction times for a single dataset (~300–500 brain voxels) were 0.022 ± 0.004 s (RF; mean ± standard deviation), 0.094 ± 0.010 s (CNN), 0.119 ± 0.017 s (CNN + RF), 0.169 ± 0.055 s (ICNN), and 0.205 ± 0.150 s (ICNN+RF), respectively. Of these models, the CNN achieved the highest AUC (0.982 ± 0.004). Sample voxels that were correctly and incorrectly predicted using the CNN are shown in Figure 3. Compared to a typical good spectrum (Figure 3A), spectra that were incorrectly predicted “bad” often exhibited wide peaks and significant frequency shifts (Figure 3B, left). Voxels that were incorrectly predicted “good” may exhibit multiplets in the spectral peaks (Figure 3B, middle) or low SNR and lipid contamination in the spectra (Figure 3B, right).

**Table 1:** Model training time, AUC scores, AUC-PR scores, and accuracy results.

Dataset	Model	Training time (s)	AUC	AUC-PR	Accuracy
*ND*	RF	64 ± 2	0.974 ± 0.006	0.971 ± 0.009	0.910 ± 0.016
	**CNN**	**158 ± 4**	**0.982 ± 0.004**	**0.985 ± 0.005**	**0.928 ± 0.015**
	CNN + RF	160 ± 3	0.977 ± 0.006	0.975 ± 0.011	0.926 ± 0.012
	ICNN	195 ± 38	0.981 ± 0.004	0.984 ± 0.005	0.926 ± 0.011
	ICNN + RF	199 ± 38	0.972 ± 0.004	0.975 ± 0.011	0.926 ± 0.012
*BT*	RF	**16 ± 0**	**0.976 ± 0.016**	**0.881 ± 0.069**	**0.920 ± 0.060**
	**CNN**	52 ± 1	0.970 ± 0.016	0.982 ± 0.007	0.930 ± 0.026
	CNN + RF	54 ± 1	0.965 ± 0.023	0.984 ± 0.014	0.932 ± 0.024
	ICNN	86 ± 7	0.967 ± 0.017	0.986 ± 0.010	0.914 ± 0.024
	ICNN + RF	88 ± 7	0.964 ± 0.019	0.975 ± 0.053	0.926 ± 0.029
*BT—evaluated in T2L voxels*	RF	-	0.969 ± 0.020	0.888 ± 0.139	0.830 ± 0.239
	**CNN**	**-**	**0.973 ± 0.018**	**0.976 ± 0.021**	**0.912 ± 0.041**
	CNN + RF	-	0.972 ± 0.139	0.977 ± 0.012	0.918 ± 0.022
	ICNN	-	0.965 ± 0.019	0.975 ± 0.030	0.890 ± 0.047
	ICNN + RF	-	0.963 ± 0.026	0.954 ± 0.077	0.908 ± 0.045

Mean and standard deviation values are based on 10 separate runs with randomly selected training, validation, and test sets. The bold values represent the ones with the highest AUC.

### The BT dataset

The model training times, AUC, AUC-PR, and accuracy values are given in Table 1, and sample ROC for all models along with CNN and ICNN training/validation loss and accuracy curves are illustrated in Figure 2B. Average prediction times for a single dataset of size (~300–500 brain voxels) were 0.021 ± 0.003 s (RF), 0.102 ± 0.010 s (CNN), 0.132 ± 0.018 s (CNN + RF), 0.174 ± 0.074 s (ICNN), and 0.255 ± 0.184 s (ICNN+RF), respectively. As with the ND data, all models performed well. The RF achieved the highest AUC (0.976 ± 0.016); however, when evaluated only in T2L voxels, the CNN achieved the highest AUC (0.973 ± 0.018). Additionally, the AUC-PR and accuracy of the CNN and both hybrid models were higher than that of the RF.

**Figure 4:**
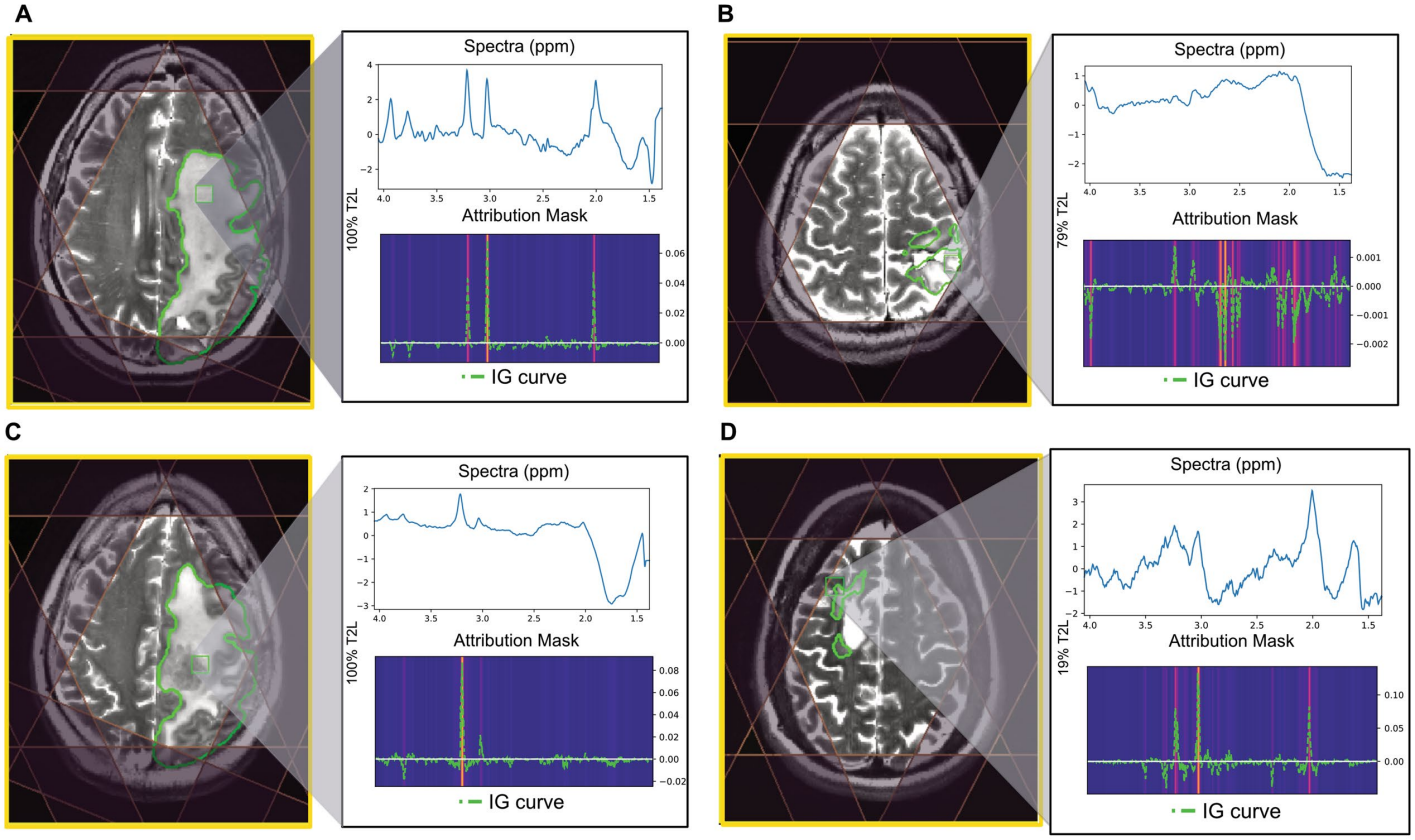
Examples of tumor voxels for patients in the BT dataset using CNN trained with leave-one-out validation. T2 FSE image shown with MRSI prescription saturation bands, T2L outlined in green for each example, and the location of the highlighted voxel. The attribution mask and overlayed IG curve for each of the highlighted voxels are shown below the spectra. **(A)** Voxel correctly predicted as “good” (100% T2L). **(B)** Voxel correctly predicted as “bad” (79% T2L). **(C)** Voxel labeled “good” but predicted as “bad” using CNN (100% T2L). **(D)** Voxel labeled “bad” but predicted as “good” using CNN (19% T2L).

Using the CNN, examples of correctly and incorrectly predicted T2L voxels are shown in Figure 4 along with their spectra, IG curves, and attribution masks. The voxels correctly predicted as “good” typically exhibited prominent *N*-acetyl-aspartate (NAA), choline, and creatine peaks, which strongly influenced the CNN prediction as demonstrated by the IG curve and attribution mask (Figure 4A). Voxels correctly predicted “bad” either lacked these peaks or demonstrated greater dispersion in the spectral importance as seen in the attribution mask (Figure 4B). The voxel highlighted in Figure 4C lacks a prominent NAA peak along with an elevated Cho peak and was incorrectly predicted as “bad.” The attribution mask for this voxel demonstrates how this spectral location for NAA did not strongly influence the prediction, as is typical of “good” voxels. Finally, the voxel in Figure 4D was incorrectly labeled “good” and its attribution mask indicates strong influences in spectral locations for choline and creatine.

## Discussion

To adopt 3D MRSI into routine clinical practice, careful inspection of spectra quality is required before interpreting metabolic maps from quantification results. This study examined the performance of several ML models to rapidly and automatically label spectra data. In contrast to previous studies, clinical datasets with varying quality and disease were used to train and evaluate ML models. The inclusion of various disease types in the ND dataset helped to create a more balanced training set. The models evaluated included a random forest classifier and two deep learning convolutional neural network models (CNN, and ICNN) as well as their hybrid models (CNN + RF and ICNN + RF).

All ML models performed exceptionally well, achieving AUCs of at least 0.964, AUC-PR of at least 0.881, and accuracies of at least 0.910. These results demonstrate such methodologies can be readily implemented in clinical MRSI processing workflows. Because of the aberrant metabolism seen in tumor lesions and resulting atypical spectra, we chose to separate the data into the ND and BT datasets. For both datasets, the simple RF classifier produced similar AUC compared to the more complex models. However, comparing metrics such as AUC-PR and prediction accuracies, the CNN and hybrid classifiers outperformed the RF. Overall, prediction accuracies for the ND dataset were higher than the BT dataset. This was, in part, due to the difference in training sizes. Together, these results suggest that the more complex models may require both more comprehensive training sets and further refinement of network architecture.

In a 3D whole brain MRSI dataset with 1 cc spatial resolution, the number of voxels within the brain is about 300–500. In this study, expert raters estimated the process of labeling spectral quality of all voxels in a single dataset took at least 10–15 min. With the application of the ML approaches, labeling for all voxels of a single examination was performed in under a second with an AUC of at least 0.964 for all models evaluated. This dramatic reduction in labeling time would allow for on-the-fly processing to extract quantitative metabolite concentrations and thus provide rapid feedback to the clinical team.

Overall, spectral quality mispredictions with ML methods could be due to several influencing factors. As seen in Figure 3, spectra with significant frequency offsets were mispredicted, as were voxels that with wide spectral peaks and low SNR. The IG curves and attribution masks allowed us to more specifically explore areas of the spectra which influence CNN model predictions. The CNN model predictions appeared to be heavily influenced by spectral peaks for choline, creatine, and NAA. In the BT dataset, attribution masks for voxels with incorrect QC classifications underscore the importance of preprocessing steps such as baseline correction and phase and frequency correction, which can distort spectra causing spurious peaks that bias ML predictions. The tissue heterogeneity exhibited in tumor voxels (i.e., elevated choline coupled with a decrease in NAA) may also result in mispredictions, as seen in Figure 4C.

It is important to note that the ML classification was evaluated in a conservative manner as voxels were labeled as “good” only if both raters labeled it as “good” and were labeled as “bad” if either rater labeled it as “bad.” Classification results are expected to improve with a training set built with a third tie-breaking rater that minimizes subjective bias. In the future, a three-class model may also be explored in which voxels are classified as “good,” “bad,” or “uncertain.” A human-ML hybrid framework in which an expert classifier manually examines only voxels for which a ML classifier predicted the “uncertain” label may be considered as a middle-ground between automated and manual QC. With the current models, additional automated filtering of voxels based on set SNR or peak width criterion may help account for voxels with B1 inhomogeneity or chemical shift misregistration errors. Optimization of model hyperparameters is also expected to improve ML-based QC. Although the ND training dataset was relatively balanced (54:46 for “good”:“bad” voxels), the BT dataset was comparatively less balanced (71:29). Thus, the accuracy of models trained for brain tumor data is expected to improve either via the use of transfer learning (initializing models with parameters trained using the ND data), or, as noted above, with the availability of a larger brain tumor training dataset more representative of abnormal metabolism exhibited in tumor spectra. Complex network architectures that have shown success with labeling of 1D data, such as the bi-directional LSTM-CNN hybrid models (Zhu et al., 2019), may also be explored for this data. Finally, the addition of anatomical information may be explored using a 4D neural network (3 spatial and 1 spectral dimension), to filter out B1 inhomogeneities and chemical shift misregistration. However, such models may be considerably more computationally intense compared to the 1D networks evaluated here.

## Conclusion

The ability of ML methods to predict spectral quality was evaluated on 3D MRSI datasets acquired from healthy volunteers, patients with neurological disorders, and patients with brain tumors. A 6-layer CNN and a simple RF classifier produced high AUC for determining quality of data from neurological and brain tumor patients. The models have the appeal of both simplicity and performance that is comparable to more complex architectures which performed similarly.

## Data availability statement

The models and example data will be made available by the authors. Further inquiries can be directed to the corresponding author.

## Ethics statement

The studies involving humans were approved by Institutional Review Board of University of California San Francisco. The studies were conducted in accordance with the local legislation and institutional requirements. The participants provided their written informed consent to participate in this study.

## Author contributions

YL contributed to the conception and design of the study. EX, HL, SV, and YL setup processing scripts. DX and YL labeled spectra data quality. SV, HL, and EX performed data analysis. HR, MS, and JL provided suggestions on methodology. SV, DX, and YL wrote the first draft of the manuscript. All authors contributed to the article and approved the submitted version.

## Funding

This study was supported by NIH R21 HD092660, R01 CA273028, and R01 CA262630.

## Conflict of interest

The authors declare that the research was conducted in the absence of any commercial or financial relationships that could be construed as a potential conflict of interest.

## Publisher’s note

All claims expressed in this article are solely those of the authors and do not necessarily represent those of their affiliated organizations, or those of the publisher, the editors and the reviewers. Any product that may be evaluated in this article, or claim that may be made by its manufacturer, is not guaranteed or endorsed by the publisher.
